# Use of Injection of Hemostatic Gelfoam Mixture During Percutaneous Core Biopsy for Renal Tumors: A Comparative Retrospective Study of Outcomes Regarding Bleeding Complications, Hospital Stay, and Diagnostic Yield Accuracy

**DOI:** 10.3390/diagnostics15070836

**Published:** 2025-03-25

**Authors:** Antonios Michailidis, Georgia Mingou, Eleni Tsakirmpaloglou, Panagiotis Kosmoliaptsis, Danae Makri, Ioannis Papadimitriou, George Dimou, Christos Giankoulof, Evangelos Petsatodis

**Affiliations:** Interventional Radiology Department, “Georgios Papanikolaou” General Hospital of Thessaloniki, 57010 Exochi, Greecedanaemakri@hotmail.com (D.M.); vpetsatodis@hotmail.com (E.P.)

**Keywords:** percutaneous kidney biopsy, renal mass biopsy, Gelfoam, hemorrhagic complications, renal cancer, hospital stay reduction, diagnostic yield

## Abstract

**Background/Objectives:** Percutaneous kidney biopsy (PKB) is a valuable diagnostic tool for evaluating renal masses and suspected renal cancer but carries a risk of hemorrhagic complications. This study aimed to determine whether injecting a hemostatic Gelfoam mixture into the biopsy tract reduces post-procedural bleeding while maintaining diagnostic accuracy. **Methods:** This retrospective study included 500 patients who underwent PKB at our hospital between 2019 and 2024. Patients were equally divided into two groups: Group A (*n* = 250) received Gelfoam injection into the biopsy tract, and Group B (*n* = 250) underwent standard PKB without Gelfoam. Hemorrhagic complications were categorized as mild, mild–moderate, moderate, or severe based on immediate and 4-h post-procedure CT findings. Management protocols included same-day discharge for mild cases (with next-day re-evaluation) and 24-h observation for mild–moderate cases. **Results:** Group A had significantly fewer moderate–severe hemorrhages compared to Group B (1.3% vs. 4.0%, *p* = 0.034) and a higher rate of same-day discharge (84% vs. 40%, *p* < 0.05). These differences led to a notable reduction in total hospitalization days (43 vs. 167) and decreased overall costs. Diagnostic yield was similarly high in both groups (98.5% vs. 97.8%, *p* = 0.72). **Conclusions:** Gelfoam injection during PKB effectively reduces hemorrhagic complications and shortens hospital stay without compromising diagnostic accuracy. Routine use of Gelfoam—especially in high-risk patients—is supported by these findings, and further prospective studies are recommended to validate these results.

## 1. Introduction

Percutaneous kidney biopsy (PKB) has emerged as a cornerstone in the evaluation of renal masses, including suspected renal cell carcinoma (RCC). This minimally invasive procedure provides critical histopathological information for accurate tumor characterization, staging, and therapeutic decision-making, thereby reducing the need for more invasive surgical interventions [1,2]. Despite its established utility, PKB carries inherent risks—most notably, hemorrhage ranging from self-limited hematuria to life-threatening perinephric hematomas requiring urgent intervention [3,4].

Bleeding complications following renal biopsy in patients with renal tumors are particularly concerning, with reported rates varying widely depending on patient-specific risk factors. Patients with preexisting conditions such as coagulopathies, advanced age, or those with larger or more vascular tumors may experience significantly higher rates of hemorrhage. These findings underscore the importance of selecting an appropriate hemostatic method to mitigate bleeding complications in high-risk groups [3].

Minimizing hemorrhagic complications is paramount. To this end, several strategies have been implemented, including the use of smaller gauge needles, real-time imaging guidance (ultrasound or computed tomography), strict patient selection, and meticulous periprocedural optimization of coagulation parameters [5]. Nonetheless, the kidney’s extensive vascular network and high perfusion pressure continue to pose challenges, especially for patients with hypertension, chronic kidney disease, or inherited coagulopathies [6,7].

In recent years, the use of local hemostatic agents has gained attention as a means to further reduce post-biopsy bleeding. One promising agent is Gelfoam (Pfizer, New York, NY, USA), an absorbable gelatin sponge that has been effectively used in gastrointestinal, hepatic, and thoracic interventions [8,9,10]. Gelfoam works by providing a matrix that promotes platelet aggregation and thrombosis, forming an immediate mechanical barrier when injected into the biopsy tract. This technique has already shown benefits in lung biopsies by significantly reducing the incidence of pneumothorax and hemorrhage [5,11,12,13]. Other commonly used hemostatic agents are thrombin-based matrices, and fibrin sealants (like Floseal (Baxter, Deerfield, IL, USA) or fibrin glue) are further analyzed in the discussion section of the manuscript.

However, despite these promising applications, the adoption of Gelfoam in PKB has been tempered by limitations in previous studies. Many of these investigations were constrained by small sample sizes, retrospective designs, and limited follow-up periods, leaving several questions about their efficacy and safety unanswered. In parallel, while alternative hemostatic agents—such as fibrin sealants and thrombin-based agents—have been explored, they often present challenges regarding ease of use, cost-effectiveness, and adaptability to the renal biopsy setting. Gelfoam, by contrast, offers a favorable balance of these factors, making it an attractive option for standardizing PKB procedures [14,15,16,17].

Additionally, concerns have been raised about whether Gelfoam injection might compromise the diagnostic yield by dislodging tissue specimens or contaminating biopsy cores. Nevertheless, accumulating evidence suggests that these risks are minimal and that the benefits in reducing hemorrhagic complications outweigh such concerns [16,17].

An important consideration is the timing of hemorrhagic events post-PKB. While some patients experience bleeding immediately after the procedure, others develop delayed hemorrhage hours later, necessitating extended observation and hospitalization. Clarifying these patterns is crucial for optimizing post-procedure care and evaluating whether interventions like Gelfoam tract embolization can facilitate timely discharge and reduce healthcare expenditures [18,19,20,21].

Given these considerations, our study systematically evaluates whether injecting Gelfoam into the biopsy tract during PKB for suspected RCC reduces post-procedural hemorrhagic complications, shortens hospital stays, and maintains a high diagnostic yield. By retrospectively analyzing 250 patients who received Gelfoam tract embolization compared to 250 historical controls, we aim to address the limitations of prior studies and provide robust evidence supporting the routine use of this technique—especially in high-risk cases. Our findings are intended to refine patient selection and post-biopsy monitoring protocols, contributing to the broader literature on the safety and efficacy of local hemostatic agents in interventional radiology [20,21,22,23].

## 2. Materials and Methods

### 2.1. Study Design

This retrospective study analyzed data from 500 patients who underwent renal mass biopsy at our hospital between January 2019 and January 2024. The imaging data used in this study were retrospectively extracted from the clinical records, and the study design did not involve a prospective intervention protocol. The study was exempt from Institutional Review Board approval (exemption decision protocol number: 112758-4). Ethics Statement: The ethical implications of retrospective data collection were carefully reviewed and managed in accordance with institutional guidelines. Patients were divided equally into two groups:

- Group A (Gelfoam): Patients received an injection of a Gelfoam slurry into the biopsy tract immediately following tissue sampling.
- Group B (Control): Patients underwent standard PKB without Gelfoam injection.

### 2.2. Patient Selection

#### 2.2.1. Inclusion Criteria

- Adult patients (≥18 years).
- Underwent PKB for the evaluation of suspected renal cancer.
- Procedures that included exactly 2 biopsy samples using 2 tissue passes with an 18-gauge biopsy pistol.
- Availability of complete clinical data (demographics, biopsy indications, procedural details, outcomes).

#### 2.2.2. Exclusion Criteria

- Patients with known coagulopathies or those on anticoagulant therapy not managed per protocol.
- Incomplete or missing procedural data.
- Cases with more than 2 biopsy samples or multiple tissue passes.
- While most tumors measured 4.3–4.5 cm, no size-based exclusion was applied; patients on antiplatelet medications were managed per institutional guidelines.

#### 2.2.3. Patient Allocation

The two groups were defined based on a change in our institution’s standard clinical practice regarding renal biopsy. Prior to January 2022, our standard protocol did not include the injection of a hemostatic Gelfoam slurry into the biopsy tract (control group). Beginning in January 2022, our protocol was updated to routinely include Gelfoam injection after tissue sampling. Therefore, patients who underwent PKB from January 2019 to December 2021 comprise Group B (Control: standard PKB without Gelfoam injection), and those from January 2022 to January 2024 comprise Group A (Gelfoam: PKB with an injection of Gelfoam slurry into the biopsy tract immediately following tissue sampling). No other significant changes were made to our methodology, thus maintaining the retrospective nature of the study and minimizing bias.

### 2.3. Biopsy Procedure

All procedures were performed under CT guidance by experienced interventional radiologists using a coaxial needle system. Tissue samples were obtained with an 18-gauge biopsy needle, ensuring at least 2 cm of material was collected over two passes.

#### 2.3.1. Gelfoam Preparation and Injection

**Figure 1:**
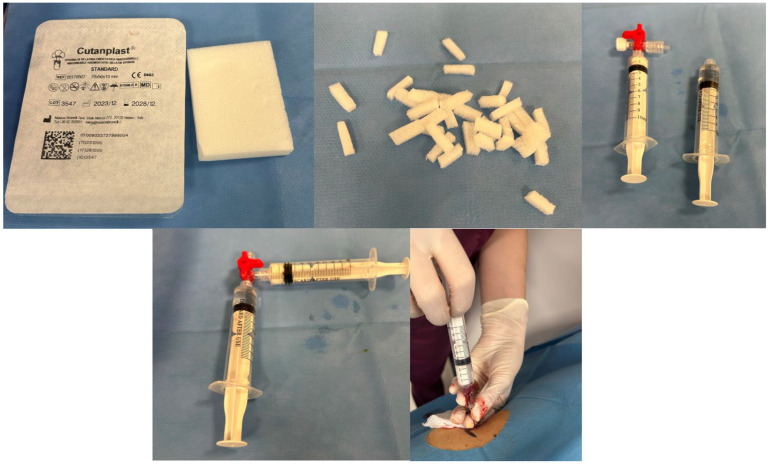
Two 10-mL luer lock syringes coupled to a three-way stopcock were used in order to mix the gelatin sponge (cut into small pieces of approximately 2 to 3 mm) into saline until it formed a paste. Then, 2–4 mL of the paste was administered through the coaxial needle, along the path of the biopsy, from the target site to the renal capsule [5].

**Figure 2:**
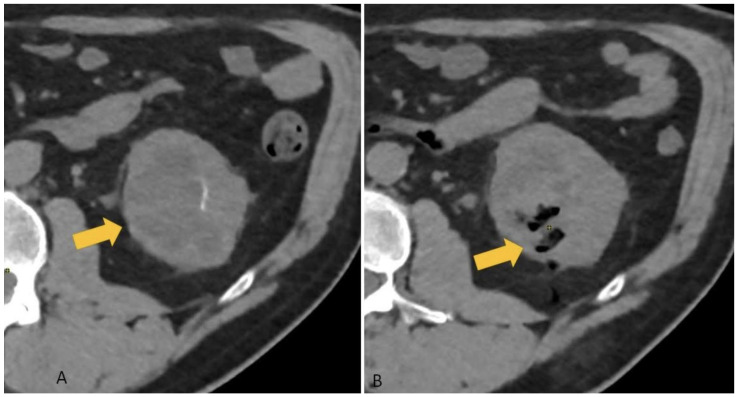
(**A**) Suspected renal mass (yellow arrow)—target to CT-guided percutaneous biopsy, (**B**) post-biopsy non-contrast CT scan, showing the dissemination of the gelatin foam paste along the site of the biopsy to the renal capsule.

Two 10-mL luer lock syringes connected via a three-way stopcock were used to mix small pieces (approximately 2–3 mm) of gelatin sponge with sterile saline until a smooth paste formed. Approximately 2–4 mL of this slurry was then injected through the coaxial needle (see Figure 1) along the biopsy tract—from the target lesion to the renal capsule (see Figure 2) [5].

#### 2.3.2. Imaging and Post-Procedure Monitoring

**Figure 3:**
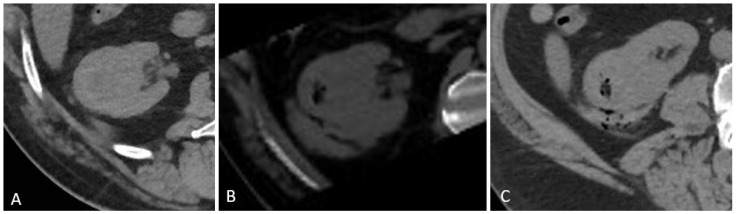
(**A**) Suspected renal mass—target to CT-guided percutaneous biopsy, (**B**) post-biopsy non-contrast CT scan, showing the dissemination of the gelatin foam paste as well as a small perirenal hematoma, which remained stable at the 4 h follow-up non-contrast CT (**C**).

At the end stage of the procedure, as per standard, renal biopsy protocol, all patients underwent a non-contrast CT scan to assess for hemorrhagic complications. A follow-up CT was performed 4 h later to detect any late-onset bleeding (see Figure 3) (this imaging protocol is part of our routine clinical practice and reflects our retrospective review). All patients underwent CT imaging to promptly identify both immediate and early delayed hemorrhagic complications. Although literature suggests that delayed bleeding may occur beyond 24 h, additional imaging beyond 4 h was performed only if clinically indicated, thereby balancing diagnostic sensitivity with resource utilization and radiation minimization. Patients were monitored for a minimum of 6 h post-procedure and after that, depending on the complications:

- Patients with **mild hemorrhage** (small perirenal fluid without an organized hematoma) were discharged with next-day clinical and laboratory evaluation.
- Patients with **mild–moderate hemorrhage** (hematoma < 3 cm in diameter) were admitted for at least 24 h of observation, with further clinical and laboratory evaluations at 12-h intervals.
- Patients with **moderate to severe hemorrhage** (hematoma > 3 cm or with extension/contrast extravasation) were promptly evaluated by both a surgeon and the interventional radiologist for further management.

#### 2.3.3. Pre-Biopsy Assessment

Although a formal pre-biopsy renal bleeding risk assessment tool was not utilized, all patients underwent a standard clinical evaluation—including a review of coagulation parameters and overall bleeding risk—as part of routine care.

### 2.4. Data Collection

Data were retrospectively obtained from the hospital’s electronic medical records. Variables included patient demographics, biopsy indications, procedural details (including Gelfoam use), and post-procedural outcomes. The primary outcome was the incidence of hemorrhagic complications, while secondary outcomes included length of hospital stay, diagnostic yield (defined as the ability to obtain adequate tissue for histopathology), and cost analysis.

### 2.5. Statistical Analysis

Data analysis was performed using IBM SPSS version 27.0 (IBM Corp., Armonk, NY, USA). Continuous variables were tested for normality using the Shapiro–Wilk test and were reported as means (SD) or medians (IQR) as appropriate. Categorical variables were presented as frequencies and percentages. Comparisons between groups were performed using the Chi-square test for categorical data and the Student’s t-test or Mann–Whitney U test for continuous data. A *p*-value < 0.05 was considered statistically significant.

### 2.6. Classification of Hemorrhagic Complications

**Figure 4:**
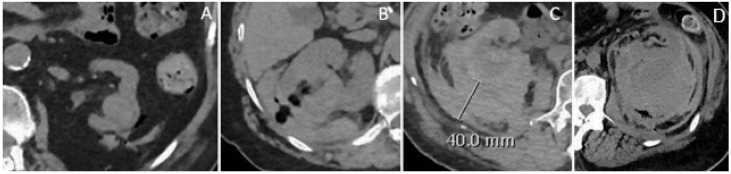
Classification of Hemorrhaging Complications. Hemorrhagic complications were categorized as mild, mild-moderate, moderate, and severe. (**A**) Mild hemorrhage: small perirenal fluid—not organized hematoma. (**B**) Mild-moderate hemorrhage: perirenal hematoma with maximum transverse diameter < 3 cm. (**C**) Moderate hemorrhage: large perirenal hematoma with maximum transverse diameter > 3 cm or with extension to retroperitoneum. (**D**) Severe hemorrhage: extensive perirenal hematoma with possible capsular rupture and contrast extravagation.

Bleeding was categorized (Figure 4) per a modified CIRSE (Cardiovascular and Interventional Radiological Society of Europe)-based system (Table 1):

- **Mild:** Minimal perirenal fluid, no organized hematoma.
- **Mild–moderate:** Hematoma with a maximum transverse diameter < 3 cm.
- **Moderate:** Hematoma > 3 cm or extension into the retroperitoneum.
- **Severe:** Extensive hematoma with potential renal capsule rupture and contrast extravasation.

Notably, while mild–moderate cases warranted 24-h observation, they were distinct from CIRSE Grade 3 (or higher) complications that require immediate intervention [6,15].

## 3. Results

### 3.1. Patient Demographics and Clinical Characteristics

A total of 500 patients were included (250 per group). The mean age was 52.4 years (SD ± 15.6) with a slight male predominance (56% in Group A and 58% in Group B; *p* = 0.54). Baseline characteristics, including mean tumor size (4.3 ± 1.1 cm in Group A vs. 4.5 ± 1.3 cm in Group B; *p* = 0.34) and PADUA (Preoperative Aspects and Dimensions Used for an Anatomical) renal score (8.2 ± 2.1 vs. 8.0 ± 1.9; *p* = 0.52), were similar between the groups (Table 2).

### 3.2. Incidence of Hemorrhagic Complications

Bleeding complications were evaluated immediately post-procedure and at 4 h, with findings re-categorized as needed:

- Group A (Gelfoam):
  ⭘ Mild hemorrhage: 40% (100/250)
  ⭘ Mild–moderate hemorrhage: 14.8% (37/250)
  ⭘ Moderate hemorrhage: 1.3% (3/250)
  ⭘ Severe hemorrhage: 0%
- Group B (No Gelfoam):
  ⭘ Mild hemorrhage: 48% (120/250)
  ⭘ Mild–moderate hemorrhage: 56% (140/250)
  ⭘ Moderate hemorrhage: 3.6% (9/250)
  ⭘ Severe hemorrhage: 0.4% (1/250; required embolization)

In Group A, only 20% of patients initially classified as having mild hemorrhage required reclassification to mild–moderate (leading to one day of hospital observation), compared to 50% in Group B (note that the 20% reclassification rate in Group A reflects the proportion of mild cases needing additional observation and does not represent the overall bleeding rate). Overall, significant (moderate–severe) complications were observed in 1.3% of Group A versus 4% in Group B (*p* = 0.034) (Table 3).

### 3.3. Diagnostic Yield

The diagnostic yield was high and comparable between both groups: 98.5% in Group A and 97.8% in Group B (*p* = 0.72). No cases in either group were rendered non-diagnostic due to insufficient tissue.

### 3.4. Subgroup Analysis

A subgroup analysis evaluating potential predictors of hemorrhage—such as patient age, biopsy indication, and number of tissue passes—revealed no significant correlations. Patients with hypertension, however, exhibited a trend toward a higher risk of bleeding (*p* = 0.08). This non-significant trend may be attributed to the relatively small sample size within the hypertensive subgroup and variability in the severity of hypertension.

### 3.5. Hospital Stay and Cost Analysis

**Figure 5:**
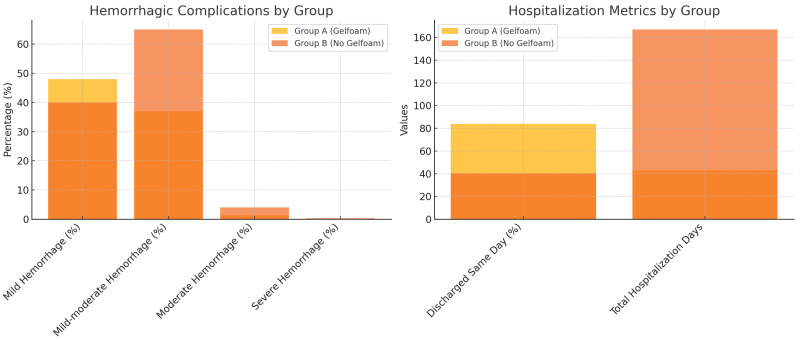
**Hemorrhagic Complications by Group**: Displays the distribution of mild, mild-moderate, moderate, and severe hemorrhagic complications between Group A (Gelfoam) and Group B (No Gelfoam). **Hospitalization Metrics by Group**: Compares the total hospitalization days and the percentage of patients discharged the same day for each group.

The mean hospital stay was significantly shorter in Group A (0 ± 1 day) compared to Group B (1 ± 1 day; *p* < 0.01). In total, Group A accounted for 43 hospitalization days versus 167 days in Group B (Figure 5). With an estimated cost of $120 per hospital day, the cost saving was approximately $14,800. Cost estimations were based on the reduction in hospitalization days, while the cost of the Gelfoam system (approximately $4 per patient or $1000 total for 250 patients) was minimal in comparison. The net savings were thus substantial.

## 4. Discussion

### 4.1. Overview of Findings and Clinical Significance

The key finding of this study is that injecting a Gelfoam slurry into the biopsy tract significantly mitigates the risk of hemorrhagic complications after percutaneous kidney biopsy (PKB) for suspected renal cell carcinoma. Compared to the control group, the Gelfoam cohort exhibited markedly lower rates of moderate–severe hemorrhage (1.3% vs. 4.3%, *p* = 0.034), a higher same-day discharge rate (84% vs. 40.4%), and substantially fewer overall hospitalization days (43 vs. 167). These results align with the mechanistic rationale that Gelfoam acts as both a physical barrier and pro-thrombotic scaffold when placed in the biopsy tract.

### 4.2. Comparing Gelfoam Tract Embolization in Other Procedures

The practice of utilizing hemostatic agents, particularly Gelfoam, is not novel and has seen widespread use in other organs. In thoracic interventions, for instance, the application of Gelfoam to seal the biopsy tract has consistently been shown to reduce pneumothorax rates and bleeding risks [5,11,22]. Similarly, hepatic biopsies and tumor ablations often employ Gelfoam pledgets or slurry to minimize hemorrhage [9,10]. These cross-procedural parallels reinforce the adaptability and efficacy of Gelfoam as a reliable adjunct across different anatomic sites.

In the context of renal procedures, however, its use has not been universally adopted. One possible explanation is that kidney biopsies are often performed with the assumption that smaller needles, real-time imaging, and appropriate patient selection alone can keep bleeding rates within acceptable limits. Yet, as many interventionalists know, certain renal lesions—particularly those that are hypervascular, centrally located, or in patients with baseline hypertension—carry a higher risk for post-biopsy complications [22,23]. Our findings suggest that in such high-risk scenarios, the addition of Gelfoam tract embolization can significantly improve patient outcomes, echoing evidence from more recent studies.

### 4.3. Comparing Gelfoam to Other Hemostatic Agents

The evaluation of different hemostatic agents for renal biopsy reveals that while Gelfoam remains the cornerstone due to its cost-effectiveness, ease of use, and proven efficacy as a physical barrier and pro-thrombotic scaffold, alternative agents such as thrombin-based matrices and fibrin sealants offer distinct advantages that may be particularly beneficial in high-risk scenarios. Gelfoam’s widespread adoption [24] is underscored by its ability to significantly reduce hemorrhagic complications—with studies demonstrating lower moderate-to-severe bleeding rates and shorter hospitalization durations—while its straightforward preparation and application via coaxial needles make it a practical option for routine procedures. Conversely, thrombin-based agents, which integrate gelatin sponges with active thrombin to accelerate clot formation, provide a more rapid and reliable hemostatic effect, especially in patients with coagulopathies or hypervascular lesions, albeit at a higher cost that limits their universal application. Fibrin sealants, by mimicking the final step of the coagulation cascade to produce a robust adhesive clot, represent the most potent option; however, their complex preparation, expense, and potential logistical challenges hinder their routine use in percutaneous kidney biopsies. Together, these findings highlight the importance of tailoring hemostatic strategy to individual patient risk profiles and procedural nuances, suggesting that while Gelfoam tract embolization is highly effective and economical for many patients, selective use of thrombin-based agents or fibrin sealants may further optimize outcomes in cases where conventional measures might be insufficient. Future prospective studies are warranted to refine patient selection criteria and to compare the long-term cost-effectiveness and diagnostic yield associated with these diverse hemostatic approaches.

### 4.4. Implications for Observation Strategies and Cost Reduction

A defining element of our institutional protocol is the systematic 4-h follow-up CT scan to catch delayed hemorrhages, followed by 24-h observation for those with mild–moderate or moderate bleeds. The fact that delayed hemorrhage can occur even after initially benign imaging findings is well documented, with some studies citing an 8–33% rate of significant bleeding manifesting after the first few hours [18,19]. Our study underscores the value of vigilant post-biopsy imaging. However, a widespread challenge is balancing patient safety with the operational cost of extended hospital stays.

The data show that Group A patients had fewer complications that warranted additional observation, translating into significant cost savings without compromising safety. From a practical standpoint, these findings could argue for a more liberal application of Gelfoam, especially in “borderline” patients who may have mild coagulopathies, advanced age, or difficult tumor locations. Although Gelfoam adds a nominal cost to each procedure (approximately $4 in our practice), this is far outweighed by the savings associated with reduced hospital stays and fewer expensive interventions like angiographic embolization.

### 4.5. Diagnostic Yield Considerations

Any intervention added during biopsy must be evaluated in the context of diagnostic yield. In our series, Gelfoam usage did not hinder the pathologist’s ability to obtain an adequate sample; the yield remained above 97% in both groups. This consistency may be partly attributed to the coaxial biopsy technique, which allows for multiple tissue passes without repeatedly traversing the renal cortex. The Gelfoam slurry was injected only after tissue acquisition was complete, further minimizing any potential contamination of the core specimen. Hence, clinicians can be reassured that tract embolization with Gelfoam, when performed correctly, is unlikely to compromise pathology results.

### 4.6. Potential Limitations

Although this study spanned five years and involved 500 patients, it was retrospective in nature, limiting the ability to account for subtle confounders such as operator experience, nuanced variations in tumor vascularity, or the exact location of the renal mass (e.g., upper pole vs. lower pole or cortical vs. medullary). Prospective, randomized controlled trials with well-defined inclusion criteria for Gelfoam use could provide more definitive evidence. Additionally, most of our patients underwent exactly two biopsy passes. Whether the beneficial effects of Gelfoam might differ with a higher number of passes—or in patients with pre-existing coagulopathies—remains an open question.

Another consideration is the durability of Gelfoam’s hemostatic effect. While Gelfoam is absorbable within several weeks, its immediate efficacy appears robust. Future studies could evaluate alternative materials, such as fibrin sealants or thrombin-based agents, to determine if they confer similar or superior benefits [13,23]. The cost-effectiveness of different hemostatic materials may also warrant further comparison.

### 4.7. Future Directions

As interventional radiology continues to evolve, so too will the techniques employed to mitigate post-procedural complications. Given the promising results presented here, one logical next step is a larger, multicenter, prospective trial that directly compares Gelfoam to other hemostatic agents or even modern “plugging” devices. Standardizing the timing and frequency of follow-up imaging—whether it is 4 h, 6 h, or 24 h post-biopsy—would also enable the establishment of evidence-based guidelines for patient discharge and observation.

In parallel, further investigation is needed to identify specific patient subpopulations that derive the greatest benefit from tract embolization. For instance, does Gelfoam substantially reduce complications in patients with borderline coagulopathy or in patients who are older with multiple comorbidities? Are there particular tumor characteristics—such as central vascularity or large size—that make Gelfoam injection almost mandatory to avert complications? Answering these questions will help fine-tune patient selection and resource allocation.

### 4.8. Summary

In summary, our data provide strong support for the use of a Gelfoam slurry injection during PKB to reduce hemorrhagic complications while preserving diagnostic accuracy. By demonstrating a clear reduction in moderate–severe bleeding, fewer hospitalization days, and lower overall costs, we believe this method represents a safe and efficient approach. As healthcare systems worldwide grapple with rising expenditures, simple procedural modifications like Gelfoam tract embolization could have a meaningful impact on both patient safety and institutional economics, particularly if validated across diverse clinical settings.

## 5. Conclusions

The injection of a hemostatic Gelfoam mixture into the biopsy tract during percutaneous renal mass biopsy significantly reduces hemorrhagic complications and hospital stay without compromising diagnostic accuracy. The marked reduction in moderate–severe bleeding and the associated cost savings support the routine use of Gelfoam—especially in high-risk patients. Given the prevalence of delayed bleeding complications, the practice of 24-h observation for mild–moderate cases is justified. Future prospective studies are recommended to validate these findings and to establish standardized protocols for patient selection and post-procedural monitoring.

## Figures and Tables

**Table 1:** Below is a comparative table that maps our modified CIRSE-based classification of hemorrhagic severity to the standard CIRSE grading system provided:.

Modified CIRSE-Based Severity	Description	Approximate Standard CIRSE Grades and Descriptions
**Mild**	Minimal perirenal fluid; no organized hematoma.	**Grade 1a:** Complication during the procedure solved within the same session with the intended procedure completed.
		**Grade 1b:** Complication resolved during the session, but the intended procedure was abandoned.
**Mild–Moderate**	Hematoma with maximum transverse diameter < 3 cm.	**Grade 2:** Prolonged observation including an overnight stay (<48 h) without additional therapy or post-procedure sequelae.
**Moderate**	Hematoma > 3 cm or extension into the retroperitoneum.	**Grade 3a:** Additional post-procedure therapy or prolonged hospital stay (>48 h but <2 weeks) required; no permanent sequelae.
**Severe**	Extensive hematoma with potential renal capsule rupture and contrast extravasation.	**Grades 3b–6:** Complications requiring extended therapy or hospital stay (>2 weeks) (Grade 3b), potentially leading to permanent mild (Grade 4) or severe sequelae (Grade 5), or even death (Grade 6).

Note: This table provides an approximate comparison. Our modified system is tailored to the imaging and clinical context of renal biopsies, while the standard CIRSE classification is a broader framework used to grade complications in interventional radiology procedures.

**Table 2:** Patient Demographics and Clinical Characteristics.

Characteristic	Group A (Gelfoam, n = 250)	Group B (No Gelfoam, n = 250)	*p*-Value
Mean Age (years)	52.4 ± 15.6	51.8 ± 16.2	0.67
Gender (% Male)	140 (56%)	145 (58%)	0.54
Hypertension	140 (56%)	130 (52%)	0.21
Diabetes	50 (20%)	45 (18%)	0.48
Smoking	60 (24%)	62 (25%)	0.81
Mean Tumor Size (cm)	4.3 ± 1.1	4.5 ± 1.3	0.34
Renal Score (PADUA)	8.2 ± 2.1	8.0 ± 1.9	0.52
Biopsy Indications	250 (100%) suspected RCC	250 (100%) suspected RCC	-
Number of Biopsy Passes	2	2	-
Pre-biopsy INR	1.17 ± 0.12	1.22 ± 0.09	0.48

**Table 3:** Hemorrhagic Complications and Hospital Stay.

Outcome	Group A (Gelfoam, n = 250)	Group B (No Gelfoam, n = 250)	*p*-Value
Significant (moderate–severe) hemorrhage (%)	3 (1.3%)	10 (4.3%)	0.034
Mild hemorrhage (%)	120 (48%)	100 (40%)	<0.05
Mild–Moderate hemorrhage (%)	37 (14.8%)	140 (56%)	<0.01
Moderate hemorrhage (%)	3 (1.3%)	9 (3.6%)	0.02
Severe hemorrhage (%)	0 (0%)	1 (0.4%)	0.12
Hospital Stay (Days, Mean ± SD)	0 ± 1	1 ± 1	<0.01
Total Hospitalization Days	43	167	-
Discharged Same Day (%)	210 (84%)	101 (40.4%)	<0.05

## Data Availability

The data supporting the findings reported in this study can be accessed by contacting the authors via email. The datasets are not publicly available due to patient confidentiality, but will be provided by the authors upon reasonable request.
